# An Alignment Method Based on KF-ASMUKF Hybrid Filtering for Ship’s SINS under Mooring Conditions

**DOI:** 10.3390/s21217104

**Published:** 2021-10-26

**Authors:** Pengchao Yao, Gongliu Yang, Xiafu Peng

**Affiliations:** 1 Department of Automation, Xiamen University, Xiamen 361005, China; yaopengchao@stu.xmu.edu.cn (P.Y.); yanggongliu@buaa.edu.cn (G.Y.); 2 School of Instrumentation and Optoelectronic Engineering, Beihang University, Beijing 100191, China

**Keywords:** SINS, ASMUKF, hybrid filter algorithm, mooring alignment

## Abstract

To solve the problem that the ship’s strapdown inertial navigation system (SINS) alignment accuracy decreases with the increase of the nonlinear filtering state dimension under mooring conditions, a method based on Kalman filter (KF) and Adaptive scale mini-skewness single line sampling Unscented Kalman Filter (ASMUKF) hybrid filtering algorithm is proposed in this paper. Three improvements are made as the following: (1) adopt a new sampling strategy. To obtain the ASMUKF filtering algorithm, scale mini-skewness single line sampling is used to replaced the traditional symmetrical sampling method and an adaptive scale factor is adapted into the Unscented Kalman Filter (UKF) to correct the real-time transformation sampling process; (2) the improved ASMUKF algorithm is combined with KF to form KF-ASMUKF hybrid filtering model; (3) the hybrid filtering model is divided into linear and nonlinear parts. The linear filtering part adopts the KF filtering model and the nonlinear filtering part adopts the ASMUKF model. Then, the calculation steps of the hybrid filtering algorithm is designed in this paper. The simulation and experimental results show that the hybrid filtering algorithm proposed has certain advantages over the traditional algorithm, and it can reduce the ship’s SINS calculation amount and improve alignment accuracy under mooring conditions.

## 1. Introduction

Strapdown inertial navigation system (SINS) has the advantages of not relying on external information and good autonomy. It is one of the most important navigation methods for maritime carriers [1,2,3]. But, the error of SINS continues to expand over time, and it must be aided by other navigation methods for a long time to navigate. Thus, SINS is used to an integrated navigation system with other auxiliary equipment [4,5,6]. With the wide application of strapdown inertial navigation system on ships, the initial alignment of the strapdown inertial navigation systems plays an important role in the maneuverability of ships. For ship alignment, auxiliary external information should be used, such as a reference for position and speed information given by the global navigation satellite system [7,8,9,10]. The ship’s strapdown inertial navigation system (SINS) must complete initial alignment before entering navigation. About the problem of initial alignment, how to complete the initial alignment quickly and well under mooring conditions is the key to ensuring accurate navigation of the ship [11,12,13,14].

At present, the error propagation model and filtering algorithm are two important issues for studying the initial alignment of strapdown inertial navigation system [15]. The SINS has a large angular motion under mooring conditions. The interference angular velocity caused by the swing is much larger than the rotation angular velocity of the earth, which makes it impossible to extract the angular velocity component of the earth rotation from the gyro output, so the error model of the ship’s SINS is nonlinear [16,17,18,19]. In [20], Sun et al. proposed an inertial system initial alignment algorithm based on hidden Markov model-Kalman filter (HMM-KF). The method can suppress the high-frequency random disturbance of the initial alignment of the ship under the mooring condition and improve the azimuth accuracy under mooring condition, but the method has low alignment accuracy for large misalignment angles. In [21], Zhao et al. proposed a second-order extended Kalman filter algorithm based on second-order nonlinear measurement, which can perform initial alignment under arbitrary posture conditions. Although the method improves the alignment accuracy, it also has a large amount of calculation. In [22], Wang et al. used Extended Kalman Filter (EKF) and Unscented Kalman Filter (UKF) for initial alignment with large misalignment angles. The results show that in the case of large misalignment angles, Unscented Kalman Filter (UKF) has higher estimation accuracy than Extended Kalman Filter (EKF). But the alignment accuracy is not high in a complex environment. In [23], Gao et al. proposed an improved ACKF-KF method, which can complete the initial alignment well in the case of unknown measurement noise. But the method does not work well for the alignment of ships at sea. In [24], Yan et al. proposed an alignment algorithm based on Gauss-Hermite filter (Gauss-Hermite filter) ship strapdown inertial navigation system inertial system, without coarse alignment to quickly achieve the initial alignment of Strapdown Inertial Navigation System (SINS). However, it is difficult to determine the optimal filtering parameters of the method, which affect the alignment accuracy. In [25], Pei et al. proposed a fast initial self-alignment method for ships based on the Constrained Matrix Kalman filter (CMKF). This method can shorten the alignment time while ensuring accuracy. But the method is not effective under the condition of large misalignment angles. In [26], Rong et al. proposed an adaptive filtering ship SINS mooring alignment algorithm based on Complementary Ensemble Empirical Mode Decomposition (CEEMD), which eliminates the effect of sensor measurement noise on accuracy and improves alignment accuracy. But the amount of calculation is relatively large.

In this paper, a hybrid filter algorithm based on KF-ASMUKF is proposed. It is to solve the problem that the initial alignment accuracy of SINS decreases and the amount of calculation complexity caused by the increase of the nonlinear filter state dimension. Firstly, this paper adopts a new sampling strategy. It uses scale mini-skewness single line sampling instead of a traditional symmetrical sample. Meanwhile, introduce an adaptive scale factor into the UKF algorithm to get the ASMUKF filtering algorithm. Secondly, the improved ASMUKF filter algorithm and KF are combined to form a KF-ASMUKF hybrid filter model. Then, the hybrid filter model is decomposed into linear and nonlinear parts. The linear filter part adopts the KF filter model, the nonlinear filter part adopts the ASMUKF filter model, and the calculation steps of the hybrid filter algorithm are designed. Finally, the simulated rocking test and the mooring test are designed to verify the method’s effectiveness.

The rest of this paper is organized as follows. Section 2 defines the standard inertial coordinate system. Section 3 introduces the error model of the ship’s large misalignment strapdown inertial navigation system. Section 4 designs the improved UKF algorithm and constructs an alignment model based on the KF-ASMUKF hybrid filter algorithm and its steps. Section 5 designs simulation and offshore alignment test to verify the effectiveness of the method. Section 6 gives the conclusion.

## 2. Description of Common Coordinate System

**Figure 1:**
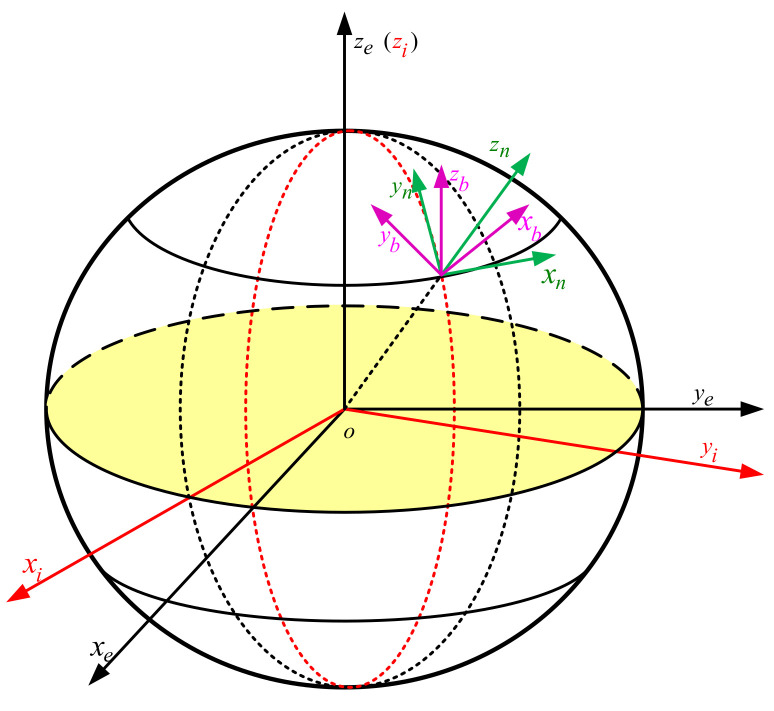
Different reference coordinate.

The different reference coordinate used in this article are shown in Figure 1, and their respective definitions are as follows:

Geocentric Inertial Coordinate System ($ox_{i}y_{i}z_{i}$.): the origin *o* is the center of the earth, $oz_{i}$ point to the rotation axis of the earth, $ox_{i}$ and $oy_{i}$ form a right-handed coordinate system with $oz_{i}$ in the equatorial plane.

Earth coordinate system ($ox_{e}y_{e}z_{e}$): the origin *o* is the center of the earth, $oz_{e}$ is along the axis of the earth, $ox_{e}$ and $oy_{e}$ are on the intersection of the equatorial plane with the prime meridian plane, forming a right-handed coordinate system with $oz_{e}$.

Navigation coordinate system ($ox_{n}y_{n}z_{n}$): the origin *o* is the center of the carrier, $oz_{n}$ points upward along the local geographic vertical line, $ox_{n}$ points east along the local latitude and $oy_{n}$ points north along the local meridian.

Carrier coordinate system ($ox_{b}y_{b}z_{b}$): the origin *o* is the center of the carrier, $ox_{b}$ points to the right along the horizontal axis of the carrier, $oy_{b}$ points forward along the longitudinal axis of the carrier, $oz_{b}$ points up along the vertical axis of the carrier.

## 3. SINS Error Model with Large Misalignment

Select the east north up (ENU) as the navigation coordinate system of the ship’s strapdown navigation system, and set its attitude differential equation at time t as:

(1) $${\dot{C}}_{b}^{n}\left(t\right)=C_{b}^{n}\left(t\right)\left(\omega_{nb}^{b}\times \right)\left(t\right)$$

$\omega_{nb}^{b}\left(t\right)$ is the projection of the angular velocity of the carrier system relative to the navigation system on the system at time t. $\omega_{nb}^{b}\times$ is the antisymmetric matrix of $\omega_{nb}^{b}$, $C_{n}^{b}\left(t\right)$ is the attitude equation of the ship carrier coordinate system relative to the navigation system at time t. According to the chain rule, the attitude equation can be decomposed into the following parts:

(2) $$C_{b}^{n}\left(t\right)=C_{n^{\prime }}^{n}\left(t\right)C_{b}^{n^{\prime }}\left(t\right)$$

Due to the existence of various errors, there is an error angle between the calculated navigation system $n^{\prime }$ of the ship carrier and the real navigation system *n*, which can be expressed as:

(3) $$\varphi =\left[\begin{array}{ccc} \varphi_{E} & \varphi_{N} & \varphi_{U} \end{array}\right]$$

The *n* coordinate system can get the $n^{\prime }$ coordinate system by rotating three times around the ENU in three directions, and its rotation matrix can be expressed as:

(4) $$C_{n}^{n^{\prime }}=\left[\begin{array}{ccc} \begin{array}{c} \cos\varphi_{N}\cos\varphi_{U}\\ -\sin\varphi_{N}\sin\varphi_{E}\sin\varphi_{U} \end{array} & \begin{array}{c} \cos\varphi_{N}\sin\varphi_{U}\\ +\sin\varphi_{N}\sin\varphi_{E}\cos\varphi_{U} \end{array} & -\sin\varphi_{N}\cos\varphi_{E}\\ -\cos\varphi_{E}\sin\varphi_{U} & \cos\varphi_{E}\cos\varphi_{U} & \sin\varphi_{E}\\ \begin{array}{c} \sin\varphi_{N}\cos\varphi_{U}\\ +\cos\varphi_{N}\sin\varphi_{E}\sin\varphi_{U} \end{array} & \begin{array}{c} \sin\varphi_{N}\sin\varphi_{U}\\ -\cos\varphi_{N}\sin\varphi_{E}\cos\varphi_{U} \end{array} & \cos\varphi_{N}\cos\varphi_{E} \end{array}\right]$$

$C_{n}^{n^{\prime }}\left(t\right)$ can be obtained according to the relationship between the equivalent rotation vector and the direction matrix:

(5) $$C_{n}^{n^{\prime }}\left(t\right)={\left(C_{n^{\prime }}^{n}\left(t\right)\right)}^{T}\approx {\left\{\left[I+\left(\varphi \times \right)\right]\left(t\right)\right\}}^{T}=\left[I-\left(\varphi \times \right)\right]\left(t\right)$$

In Formula (5), *I* is the identity matrix, φ× is the antisymmetric matrix of φ.

Formula (1) can be further decomposed into:

(6) $$\begin{array}{c} {\dot{C}}_{b}^{n}\left(t\right)=C_{b}^{n}\left(t\right)\left(\omega_{nb}^{b}\times \right)\left(t\right)\\ \;\;\;\;\;\;\;\;\;\;\;\;\;\;\;\;\;\;\;\;\;\;\;\;\;=C_{b}^{n}\left(t\right)\left[\left(\omega_{ib}^{b}-\omega_{in}^{b}\right)\times \right]\left(t\right)\\ \;\;\;\;\;\;\;\;\;\;\;\;\;\;\;\;\;\;\;\;\;\;\;\;\;=C_{b}^{n}\left(t\right)\left(\omega_{ib}^{b}\times \right)\left(t\right)-C_{b}^{n}\left(t\right)\left(\omega_{in}^{b}\times \right)\left(t\right)C_{b}^{n}\left(t\right)C_{n}^{b}\left(t\right)\\ \;\;\;\;\;\;\;\;\;\;\;\;\;\;\;\;\;\;\;\;\;\;\;\;\;=C_{b}^{n}\left(t\right)\left(\omega_{ib}^{b}\times \right)\left(t\right)-\left(\omega_{in}^{n}\times \right)\left(t\right)C_{b}^{n}\left(t\right) \end{array}$$

$\omega_{ib}^{b}$ is the angular velocity information output by the gyro. $\omega_{ib}^{b}\times$ is its antisymmetric matrix.

(7) $$\omega_{in}^{n}\left(t\right)=\omega_{ie}^{n}\left(t\right)+\omega_{en}^{n}\left(t\right)$$

(8) $$\omega_{ie}^{n}\left(t\right)={\left[\begin{array}{ccc} 0 & \omega_{ie}\left(t\right)\cos L & \omega_{ie}\left(t\right)\sin L \end{array}\right]}^{T}$$

(9) $$\omega_{en}^{n}\left(t\right)={\left[\begin{array}{ccc} \frac{V_{N}\left(t\right)}{R_{M}+h} & \frac{V_{E}\left(t\right)}{R_{N}+h} & \frac{V_{E}\left(t\right)}{R_{N}+h} \end{array}\tan L\right]}^{T}$$

In Formula (9), $V_{N}$ is the northerly velocity of the carrier in motion. $V_{E}$ is the easterly velocity. $R_{M}$ is the radius of curvature of the meridian circle. $R_{N}$ is the radius of curvature of the earth’s unitary rotation. *h* is the geographic height, and *L* is the geographic latitude.

Due to the existence of various errors in actual measurement, Formula (6) can be expressed as:

(10) $${\dot{C}}_{b}^{n^{\prime }}\left(t\right)=C_{b}^{n^{\prime }}\left(t\right)\left({\tilde{\omega }}_{ib}^{b}\times \right)\left(t\right)-\left({\tilde{\omega }}_{in}^{n}\times \right)\left(t\right)C_{b}^{n^{\prime }}\left(t\right)$$

In Formula (10), ${\tilde{\omega }}_{ib}^{b}\left(t\right)=\omega_{ib}^{b}\left(t\right)+\delta \omega_{ib}^{b}\left(t\right)$. ${\tilde{\omega }}_{in}^{n}\left(t\right)=\omega_{in}^{n}\left(t\right)+\delta \omega_{in}^{n}\left(t\right)$. $\delta \omega_{ib}^{b}\left(t\right)$ is the gyroscope measurement error at time *t*. $\delta \omega_{ib}^{b}\left(t\right)=\varepsilon^{b}$. $\delta \omega_{in}^{n}\left(t\right)$ is the calculation error of the navigation system at time *t*.

(11) $$\delta \omega_{in}^{n}\left(t\right)=\delta \omega_{ie}^{n}\left(t\right)+\delta \omega_{en}^{n}\left(t\right)$$

(12) $$\delta \omega_{ie}^{n}\left(t\right)={\left[\begin{array}{ccc} 0 & -\omega_{ie}^{n}\left(t\right)\sin L\delta L & -\omega_{ie}^{n}\left(t\right)\cos L\delta L \end{array}\right]}^{T}$$

(13) $$\delta \omega_{en}^{n}\left(t\right)=\left[\begin{array}{c} -\frac{\delta V_{N}\left(t\right)}{R_{M}+h}+\frac{\delta hV_{N}\left(t\right)}{{\left(R_{M}+h\right)}^{2}}\\ \frac{\delta V_{E}\left(t\right)}{R_{N}+h}-\frac{\delta hV_{E}\left(t\right)}{{\left(R_{N}+h\right)}^{2}}\\ \delta L\left(\omega_{ie}\left(t\right)\cos L+\frac{V_{E}\left(t\right)\sec^{2}L}{R_{N}+h}\right)+\frac{\delta V_{E}\left(t\right)}{R_{N}+h}\tan L-\frac{\delta hV_{E}\left(t\right)\tan L}{{\left(R_{N}+h\right)}^{2}} \end{array}\right]$$

Differentiate Formula (5) to get:

(14) $$\left(-\dot{\varphi }\times \right)\left(t\right)C_{b}^{n}\left(t\right)+\left(I-\varphi \times \right)\left(t\right){\dot{C}}_{b}^{n}\left(t\right)=C_{b}^{n^{\prime }}\left(t\right)\left({\tilde{\omega }}_{ib}^{b}\times \right)\left(t\right)-\left({\tilde{\omega }}_{in}^{n}\times \right)\left(t\right)C_{b}^{n^{\prime }}\left(t\right)$$

Substituting Formula (2) and Formula (6) into Formula (14) can be obtained:

(15) $$\begin{array}{c} \left(-\dot{\varphi }\times \right)\left(t\right)C_{b}^{n}\left(t\right)+\left(I-\varphi \times \right)\left(t\right){\dot{C}}_{b}^{n}\left(t\right)\\ \;\;\;\;\;\;\;\;\;\;\;\;\;\;\;\;\;\;\;\;\;\;\;\;\;\;\;\;\;\;\;\;\;\;\;\;\;\;\;\;\;\;\;\;\;\;\;\;\;\;\;\;\;\;\;\;\;\;\;\;\;\;\;\;\;\;\;\;\;\;\;\;\;\;\;\;\;\;\;\;\;\;\;\;\;\;\;\;\;=C_{b}^{n^{\prime }}\left(t\right)\left({\tilde{\omega }}_{ib}^{b}\times \right)\left(t\right)-\left({\tilde{\omega }}_{in}^{n}\times \right)\left(t\right)C_{b}^{n^{\prime }}\left(t\right)\\ \;\;\;\;\;\;\;\;\;\;\;\;\;\;\;\;\;\;\;\;\;\;\;\;\;\;\;\;\;\;\;\;\;\;\;\;\;\;\;\;\;\;\;\;\;\;\;\;\;\;\;\;\;\;\;\;\;\;\;\;\;\;\;\;\;\;\;\;\;\;\;\;\;\;\;\;\;\;\;\;\;\;\;\;\;\;\;\;=\left(I-\varphi \times \right)\left(t\right)C_{b}^{n}\left(t\right)\left[\left(\omega_{ib}^{b}+\delta \omega_{ib}^{b}\right)\times \right]\left(t\right)\\ \;\;\;\;\;\;\;\;\;\;\;\;\;\;\;\;\;\;\;\;\;\;\;\;\;\;\;\;\;\;\;\;\;\;\;\;\;\;\;\;\;\;\;\;\;\;\;\;\;\;\;\;\;\;\;\;\;\;\;\;\;\;\;\;\;\;\;\;\;\;\;\;\;\;\;\;\;\;\;\;\;\;\;\;\;\;\;\;\;\;-\left[\left(\omega_{in}^{n}+\delta \omega_{in}^{n}\right)\times \right]\left(t\right)\left(I-\varphi \times \right)\left(t\right)C_{b}^{n}\left(t\right) \end{array}$$

Multiply both sides of Formula (12) to the right by two at the same time. Ignoring the small second-order amount in the equation to get:

(16) $$\begin{array}{c} \left(\dot{\varphi }\times \right)\left(t\right)=\left[\left(\varphi \times \omega_{in}^{n}\right)\times \right]\left(t\right)+\left(\delta \omega_{in}^{n}\times \right)\left(t\right)-\left(\delta \omega_{ib}^{b}\times \right)\left(t\right)\\ \;\;\;\;\;\;\;\;\;\;\;\;\;\;\;\;\;\;\;\;\;\;\;\;\;=\left[\left(\varphi \times \omega_{in}^{n}+\delta \omega_{in}^{n}-\delta \omega_{ib}^{b}\right)\times \right]\left(t\right) \end{array}$$

The differential equation of the carrier error angle on the navigation system *n* can be expressed as:

(17) $$\dot{\varphi }\left(t\right)=(I-C_{n}^{n^{\prime }})\omega_{in}^{n}\left(t\right)+C_{b}^{n}\delta \omega_{in}^{b}\left(t\right)-C_{b}^{n}\delta \omega_{ib}^{b}\left(t\right)$$

By Formula (17) combined with Formulas (7)–(9), the attitude error component of the carrier in the (ENU) direction can be obtained:

(18) $$\begin{array}{c} {\dot{\varphi }}_{E}\left(t\right)=(\omega_{ie}\left(t\right)\sin L+\frac{V_{E}\left(t\right)\tan L}{R_{N}+h})\varphi_{N}\\ \;\;\;\;\;\;\;\;\;\;\;\;\;\;\;\;\;\;\;\;\;\;\;\;\;\;\;\;\;\;\;\;\;-\left(\omega_{ie}\left(t\right)\cos L+\frac{V_{E}\left(t\right)}{R_{N}+h}\right)\varphi_{U}-\frac{\delta V_{N}\left(t\right)}{R_{M}+h}+\frac{\delta hV_{N}\left(t\right)}{{\left(R_{M}+h\right)}^{2}}-\varepsilon_{E} \end{array}$$

(19) $$\begin{array}{c} {\dot{\varphi }}_{N}\left(t\right)=-(\omega_{ie}\left(t\right)\sin L+\frac{V_{E}\left(t\right)\tan L}{R_{N}+h})\varphi_{E}-\frac{V_{N}\left(t\right)\varphi_{U}}{R_{M}+h}\\ \;\;\;\;\;\;\;\;\;\;\;\;\;\;\;\;\;\;\;\;\;\;\;\;\;\;\;\;\;\;\;\;\;\;\;\;\;-\delta L\omega_{ie}\left(t\right)\sin L+\frac{\delta V_{E}\left(t\right)}{R_{N}+h}-\frac{\delta hV_{E}\left(t\right)}{{\left(R_{N}+h\right)}^{2}}-\varepsilon_{N} \end{array}$$

(20) $$\begin{array}{c} {\dot{\varphi }}_{U}\left(t\right)=(\omega_{ie}\left(t\right)\cos L+\frac{V_{E}\left(t\right)}{R_{N}+h})\varphi_{E}+\frac{V_{N}\left(t\right)\varphi_{N}}{R_{M}+h}\\ \;\;\;\;\;\;\;\;\;\;\;\;\;\;\;\;\;\;\;\;\;\;\;\;\;+\;\delta L\left(\omega_{ie}\left(t\right)\cos L+\frac{V_{E}\left(t\right)\sec^{2}L}{R_{N}+h}\right)+\frac{\delta V_{E}\left(t\right)\tan L}{R_{N}+h}-\frac{\delta hV_{E}\left(t\right)\tan L}{{\left(R_{N}+h\right)}^{2}}-\varepsilon_{E} \end{array}$$

The velocity equation of the carrier in the navigation system can be expressed as:

(21) $${\dot{V}}^{n}\left(t\right)=C_{b}^{n}\left(t\right)f^{b}\left(t\right)-\left(2\omega_{ie}^{n}\left(t\right)+\omega_{en}^{n}\left(t\right)\right)V_{en}^{n}\left(t\right)-g^{n}\left(t\right)$$

In the actual calculation process, due to various errors, the speed equation can be expressed as:

(22) $${\tilde{\dot{V}}}^{n}\left(t\right)=C_{b}^{n^{\prime }}\left(t\right){\tilde{f}}^{b}\left(t\right)-\left(2{\tilde{\omega }}_{ie}^{n}\left(t\right)+{\tilde{\omega }}_{en}^{n}\left(t\right)\right){\tilde{V}}_{en}^{n}\left(t\right)-{\tilde{g}}^{n}\left(t\right)$$

The velocity error equation can be obtained by Formulas (21) and (22):

(23) $$\begin{array}{cc} \delta {\dot{V}}^{n}\left(t\right) & ={\tilde{\dot{V}}}^{n}\left(t\right)-{\dot{V}}^{n}\left(t\right)\\ \; & =\left(C_{b}^{n^{\prime }}\left(t\right){\tilde{f}}^{b}\left(t\right)-C_{b}^{n}\left(t\right)f^{b}\left(t\right)\right)-\left[\left(2{\tilde{\omega }}_{ie}^{n}\left(t\right)+{\tilde{\omega }}_{en}^{n}\left(t\right)\right)\times {\tilde{\dot{V}}}^{n}\left(t\right)\right.\\ \; & \left.-\left(2\omega_{ie}^{n}\left(t\right)+\omega_{en}^{n}\left(t\right)\right)\times {\dot{V}}^{n}\left(t\right)\right]+\left({\tilde{g}}^{n}\left(t\right)-g^{n}\left(t\right)\right) \end{array}$$

In Formula (23), ${\tilde{f}}^{b}=f^{b}+\delta f^{b}$, ${\tilde{g}}^{n}=g^{n}+\delta g^{n}$, substituting Formulas (2) and (5) into Formula (23) and ignoring the second-order small quantities, we can further obtain:

(24) $$\begin{array}{c} \delta {\dot{V}}^{n}\left(t\right)=\left[\left(I-\varphi \times \right)C_{b}^{n}\left(t\right)\left(f^{b}\left(t\right)+\delta f^{b}\left(t\right)\right)-C_{b}^{n}\left(t\right)f^{b}\left(t\right)\right]\\ \;\;\;\;\;\;\;\;\;\;\;\;\;\;\;\;\;\;\;\;\;\;\;\;\;\;\;\;\;\;\;\;\;\;\;\;\;\;\;\;\;-\{\left[2\left(\omega_{ie}^{n}\left(t\right)+\delta \omega_{ie}^{n}\left(t\right)\right)+\left(\omega_{en}^{n}\left(t\right)+\delta \omega_{ie}^{n}\left(t\right)\right)\right]\times \left(V^{n}\left(t\right)+\delta V^{n}\left(t\right)\right)\\ \;\;\;\;\;\;\;\;\;\;\;\;\;\;\;\;\;\;\;\;\;\;\;\;\;\;\;\;\;\;\;\;\;\;\;\;\;\;\;\;\;-\left(2\omega_{ie}^{n}\left(t\right)+\omega_{en}^{n}\left(t\right)\right)\times V^{n}\left(t\right)+\delta g^{n}\left(t\right)\}\\ \;\;\;\;\;\;\;\;\;\;\;\;\;\;\;\;\;\;\;\;\;\;\;\;\;\;\;\;\;\;\;\approx f^{n}\left(t\right)\times \varphi +V^{n}\left(t\right)\times \left(2\delta \omega_{ie}^{n}\left(t\right)+\delta \omega_{en}^{n}\left(t\right)\right)\;\;\\ \;\;\;\;\;\;\;\;\;\;\;\;\;\;\;\;\;\;\;\;\;\;\;\;\;\;\;\;\;\;\;\;\;\;\;\;\;\;\;\;-\;\left(2\omega_{ie}^{n}\left(t\right)+\omega_{en}^{n}\left(t\right)\right)\;\;\;\times \;\delta V^{n}\left(t\right)+\;\;\delta f^{b}\left(t\right)+\;\;\;\;\delta g^{n}\left(t\right) \end{array}$$

In Formula (24), $\delta f^{b}$ is the accelerometer’s zero bias error. $\delta f^{b}=\nabla^{b}$, $\delta g^{n}$ is the gravity acceleration deviation. $\delta g^{n}\approx 0$, from the Formula (24), we can get the speed error component of the carrier in the (EN) direction at time *t*:

(25) $$\begin{array}{c} \delta {\dot{V}}_{E}\left(t\right)=\varphi_{U}f_{N}\left(t\right)-\varphi_{N}f_{U}\left(t\right)+\frac{\delta V_{E}\left(t\right)\left(V_{N}\left(t\right)\tan L-V_{U}\left(t\right)\right)}{R_{N}+h}+\delta V_{N}\left(t\right)(2\omega_{ie}\left(t\right)\sin L\\ \;\;\;\;\;\;\;\;\;\;\;\;\;\;\;\;\;\;\;\;\;\;\;\;\;\;\;\;\;\;\;\;\;\;\;\;\;\;\;\;+\frac{V_{E}\left(t\right)\tan L}{R_{N}+h})-\delta V_{U}\left(t\right)\left(2\omega_{ie}\left(t\right)\cos L+\frac{V_{E}\left(t\right)}{R_{N}+h}\right)+\delta L\left(t\right)[2\omega_{ie}\left(t\right)(V_{U}\left(t\right)\sin L\\ \;\;\;\;\;\;\;\;\;\;\;\;\;\;\;\;\;\;\;\;\;\;\;\;\;\;\;\;\;\;\;\;\;\;\;\;\;\;\;+V_{N}\left(t\right)\cos L)+\frac{V_{E}\left(t\right)V_{N}\left(t\right)}{R_{N}+h}]+\delta h\left(t\right)\frac{V_{E}\left(t\right)V_{U}\left(t\right)-V_{E}\left(t\right)V_{N}\left(t\right)\tan L}{R_{N}+h}+\nabla_{E} \end{array}$$

(26) $$\begin{array}{c} \delta {\dot{V}}_{N}\left(t\right)=-\varphi_{U}f_{E}\left(t\right)+\varphi_{E}f_{U}\left(t\right)-2\delta V_{E}\left(t\right)\left(\omega_{ie}\left(t\right)\sin L+\frac{V_{E}\left(t\right)\tan L}{R_{N}+h}\right)\\ \;\;\;\;\;\;\;\;\;\;\;\;\;\;\;\;\;\;\;\;\;\;\;\;\;\;\;\;\;\;\;\;\;\;\;\;\;\;\;\;\;\;\;\;\;\;-\frac{\delta V_{N}\left(t\right)V_{U}\left(t\right)}{R_{M}+h}-\frac{\delta V_{U}\left(t\right)V_{N}\left(t\right)}{R_{M}+h}-\delta L\left(t\right)(2V_{E}\left(t\right)\omega_{ie}\left(t\right)\cos L\\ \;\;\;\;\;\;\;\;\;\;\;\;\;\;\;\;\;\;\;\;\;\;\;\;\;\;\;\;\;\;\;\;\;\;\;\;\;\;\;\;\;\;\;\;\;\;+\frac{{V_{E}}^{2}\left(t\right)\sec^{2}L}{R_{N}+h})+\delta h\left(t\right)\left[\frac{V_{N}\left(t\right)V_{U}\left(t\right)}{{\left(R_{M}+h\right)}^{2}}+\frac{{V_{E}}^{2}\left(t\right)\tan L}{{\left(R_{N}+h\right)}^{2}}\right]+\nabla_{N} \end{array}$$

## 4. KF-ASMUKF Hybrid Filtering Algorithm

### 4.1. ASMUKF Filtering Algorithm

The traditional UKF algorithm mainly processes the statistics of the state through unscented transformation (UT), and obtains a set of sigma points according to the statistical characteristics of the state variables that need to be predicted. Then calculates the statistical factors of the sampling points after these nonlinear transformations, get the mean and covariance estimates [18]. But the traditional UKF has the problem of nonlocal sampling. This article adopts a new sampling strategy to obtain new sigma points by scaling them to solve the nonlocal sampling problem, suppress filter divergence and improve its accuracy.

The state estimation model for the nonlinear system can be expressed as:

(27) $$\begin{array}{c} x_{t}=f\left(x_{t-1}\right)+Q_{t-1}\\ y_{t}=h\left(x_{t-1}\right)+R_{t-1} \end{array}$$

In Formula (27), $x_{t}$ and $y_{t}$ represent state quantity and quantity measurement respectively. $Q_{t-1}$ and $R_{t-1}$ represent system noise and measurement noise, respectively.

New sigma sampling method:

(28) $$l_{i}=\left\{\begin{array}{cc} \bar{x} & i=0\\ \bar{x}-\left(\sqrt{P_{x}}\right)l_{i} & i=1,\cdots ,n\\ \bar{x}+\left(\sqrt{P_{x}}\right)l_{i} & i=1,\cdots ,2n \end{array}\right.$$

The weights when calculating the mean and variance are:

(29) $$\left\{\begin{array}{cc} W_{i}^{m}=\frac{W_{0}}{\alpha^{2}}+\left(1-\frac{1}{\alpha^{2}}\right), & i=0\\ W_{i}^{m}=\frac{1-W_{0}}{2^{n}\alpha^{2}},\;\;\;\;\;\; & \;\;\;\;i=1,2\\ W_{i}^{m}=\frac{2^{i-2}-W_{1}}{\alpha^{2}},\;\; & \;i=3,4,\cdots ,n \end{array}\right.$$

(30) $$\left\{\begin{array}{c} W_{i}^{c}=W_{i}^{m}+\left(1-\alpha^{2}+\beta \right),\;\;\;\;\;\;\;\;\;\;\;\;\;\;\;i=0\\ W_{i}^{c}=W_{1}^{m},\;\;\;\;\;\;\;\;\;\;\;\;\;\;\;\;\;\;\;\;\;\;\;\;\;\;\;\;\;\;\;\;\;\;\;\;\;\;\;\;\;\;\;\;\;\;\;\;\;\;\;\;\;\;\;\;\;\;\;\;\;\;\;\;\;\;\;\;\;\;\;\;\;\;\;\;\;\;\;\;\;\;i\neq 0 \end{array}\right.$$

In the Formula (30), α is the scale parameter, the value range is generally between [0, 1]. $W_{i}$ is the weight of the *i* sampling point in the minimum skewness simplex sampling. $W_{i}^{m}$ and $W_{i}^{c}$ are the sampling, respectively The weighted value used for the mean and covariance in the transformation.

(1) Time update

According to Formula (28), all sampling points can be passed:

(31) $$\zeta_{t,i}=f\left(l_{t-1,i}\right),\;\;\;\;\;i=1,2,...n$$

State prediction and its covariance can be expressed as:

(32) $${\hat{x}}_{t/t-1}=\sum_{i=0}^{2n}W_{i}^{m}\zeta_{t-1}$$

(33) $$P_{x,t/t-1}=\sum_{i=0}^{2n}W_{i}^{c}\left(\zeta_{t-1}-{\hat{x}}_{t/t-1}\right){\left(\zeta_{t-1}-{\hat{x}}_{t/t-1}\right)}^{T}+Q_{t-1}$$

(2) Measurement update

According to Formula (31), it can be concluded that all sampling points are transmitted during the measurement update process:

(34) $$\zeta_{i}=h\left(l_{i}\right),\;\;\;\;\;\;\;\;\;\;\;\;\;\;\;\;\;\;\;\;i=1,2,...2n$$

The mean, variance, and covariance of the measured information after transmission can be expressed as:

(35) $${\hat{y}}_{t}=\sum_{i=0}^{2n}W_{i}^{m}\zeta_{t,i}$$

(36) $$P_{y,t}=\sum_{i=0}^{2n}W_{i}^{c}\left(\zeta_{t,i}-{\hat{y}}_{t}\right){\left(\zeta_{t-1,i}-{\hat{y}}_{t}\right)}^{T}+R_{t}$$

(37) $$P_{xy,t}=\sum_{i=0}^{2n}W_{i}^{c}\left(\zeta_{t,i}-{\hat{x}}_{t/t-1}\right){\left(\zeta_{t,i}-{\hat{y}}_{t}\right)}^{T}$$

Then, the mean value and variance of the state quantity at time *t* can be expressed as:

(38) $$K_{t}=P_{xy,t}{\left(P_{y,t}\right)}^{-1}$$

(39) $${\hat{x}}_{t/t}={\hat{x}}_{t/t-1}+K_{t}\left(y_{t}-{\hat{y}}_{t}\right)$$

(40) $$P_{x,t/t}=P_{x,t/(t-1)}-K_{t}P_{y,t}{\left(K_{t}\right)}^{T}$$

The system estimate can be obtained as its variance, then:

(41) $$P_{t/t}=E\left[\left(x_{t/t}-{\hat{x}}_{t/t}\right){\left(x_{t/t}-{\hat{x}}_{t/t}\right)}^{T}\right]$$

Formula (41) shows that the distance between $x_{t/t}$ and ${\hat{x}}_{t/t}$. When the mini-skewness single-line sampling transformation is performed for the n time, the distance ${\hat{x}}_{t/t}$ from the sigma sampling point to the center point should satisfy $d_{n}\leq d_{t}$:

(42) $$\left\{\begin{array}{c} \alpha_{t+1/t}d_{\max}=d_{t/t}\\ d_{\max}=\max\left\{d_{i}\right\},i\in R\\ d_{i}=\sqrt{{\left(l_{i}\right)}^{T}U^{T}Ul_{i}} \end{array}\right.$$

In Formula (42), *U* is the number of sampling points. R is the index set of sampling points. According to Formula (42), the scale correction coefficient of the minimum skewness single-line sampling transformation can be calculated $\alpha_{t+1/t}$. α is corrected every time the mini-skewness single-line sampling transformation is performed. Finally, the ASMUKF algorithm can be obtained. Based on the above analysis and derivation, the flow of the ASMUKF algorithm can be derived, as shown in Table 1:

### 4.2. Mooring Alignment Model Based on KF-ASMUKF Hybrid filter

Research the ship’s mooring alignment and establish the East-North-UP(ENU) space coordinate system. Then the 11-dimensional linear state vector is:

(43) $$X\left(t\right)={\left[\begin{array}{cccc} \delta V & \varphi & \varepsilon^{b} & \nabla^{b} \end{array}\right]}^{T}$$

Combining Equations (17) and (24) can get the error equation:

(44) $$\left\{\begin{array}{c} \dot{X}\left(t\right)=f\left[X\left(t\right),t\right]+G\left(t\right)w\left(t\right)\\ Z(t)=H(t)X(t)+v(t) \end{array}\right.$$

In Formula (44), f[X(t),t] is a nonlinear state function:

(45) $$f\left[X\left(t\right),t\right]=\left[\begin{array}{c} (I-C_{n}^{n^{\prime }})\omega_{in}^{n}\left(t\right)+C_{b}^{n}\delta \omega_{in}^{b}\left(t\right)-C_{b}^{n}\delta \omega_{ib}^{b}\left(t\right)\\ \begin{array}{c} f^{n}\left(t\right)\times \varphi +V^{n}\left(t\right)\times \left(2\delta \omega_{ie}^{n}\left(t\right)+\delta \omega_{en}^{n}\left(t\right)\right)\;\\ \;-\;\left(2\omega_{ie}^{n}\left(t\right)+\omega_{en}^{n}\left(t\right)\right)\;\;\;\times \;\delta V^{n}\left(t\right)+\;\;\delta f^{b}\left(t\right)+\;\;\;\;\delta g^{n}\left(t\right) \end{array}\\ 0_{6\times 1} \end{array}\right]$$

G(t) is the control matrix of noise, w(t) is the noise matrix, where:

(46) $$G\left(t\right)=\left[\begin{array}{cc} C_{b}^{n^{\prime }} & 0_{3\times 3}\\ 0_{3\times 3} & -C_{b}^{n^{\prime }}\\ 0_{5\times 3} & 0_{5\times 3} \end{array}\right]\;\;\;\;\;\;\;\;\;\;\;\;\;\;\;\;w\left(t\right)={\left[\begin{array}{cc} \varepsilon_{w}^{b} & \nabla_{w}^{b} \end{array}\right]}^{T}$$

Observed measurement is the speed error of GPS and SINS. $H\left(t\right)=\left[{\begin{array}{cc} I_{3\times 3} & 0 \end{array}}_{3\times 8}\right]$. v(t) is the measurement noise vector

Formulas (44) and (45) show that in the nonlinear filter equations of the system, only the attitude error angle is in the nonlinear form, while the velocity error and position error appear in the system equation in linear form. Therefore, the model decomposition method can be used to decompose the filter model into a nonlinear part and a linear part. For the nonlinear part, the ASMUKF filtering model is used, and for the linear part, the KF filtering model is used. It can ensure accuracy and reduce the calculation amount of the filtering algorithm. It can also further improve the real-time performance of the algorithm.

Select the linear part state vector $X^{lin}\left(t\right)={\left[\begin{array}{ccc} \delta v^{n} & \varepsilon^{b} & \nabla^{b} \end{array}\right]}^{T}$. Using SINS to calculate the difference between the speed and the GPS output speed under the condition of static base as the measurement of the linear part. Then, the filter model after linear part discretization:

(47) $$\left\{\begin{array}{c} X^{lin}\left(t+1\right)=F^{lin}\left(t\right)X^{lin}\left(t\right)+\Xi^{lin}X^{non}\left(t\right)+G^{lin}\left(t\right)w^{lin}\left(t\right)\\ Z^{lin}\left(t+1\right)=H^{lin}\left(t+1\right)X^{lin}\left(t+1\right)+v^{lin}\left(t+1\right) \end{array}\right.$$

In Formula (47), $F^{lin}\left(t\right)$ is the transition matrix of the linear part, $F^{lin}\left(t\right)=\left[\begin{array}{cc} 0_{3\times 6} & C_{b}^{n^{\prime }}\\ 0_{6\times 3} & 0_{6\times 3} \end{array}\right]$. Ξ(t) is the input control matrix, $\Xi^{lin}={\left[\begin{array}{cc} C_{n^{\prime }}^{n}g^{n} & 0_{1\times 6} \end{array}\right]}^{T}$. $G^{lin}\left(t\right)$ is the linear input noise matrix, $G^{lin}\left(t\right)={\left[\begin{array}{cc} C_{b}^{n^{\prime }} & 0_{6\times 3} \end{array}\right]}^{T}$. $H^{lin}\left(t\right)$ is the measurement matrix of the linear part, $H^{lin}\left(t\right)=\left[\begin{array}{cc} I_{3\times 3} & 0_{3\times 6} \end{array}\right]$. $w^{lin}\left(t\right)$ is the process noise of the linear part, $w^{lin}\left(t\right)∼N\left(0,Q^{lin}\left(t\right)\right)$. $v^{lin}\left(t+1\right)$ is the measurement noise, $v^{lin}\left(t+1\right)∼N\left(0,R^{lin}\left(t+1\right)\right)$.

The nonlinear part of state equation $X^{non}\left(t\right)={\left[\varphi \right]}^{T}$ can be separated by Formula (44). The filter model after discretization of the nonlinear part:

(48) $$\left\{\begin{array}{c} X^{non}\left(t+1\right)=f^{non}\left(X\left(t\right),t\right)+\Xi^{non}{\hat{X}}^{lin}\left(t\right)+G^{non}\left(t\right)w^{non}\left(t\right)\\ Z^{non}\left(t+1\right)=H^{non}\left(t+1\right)X^{non}\left(t+1\right)+v^{non}\left(t+1\right) \end{array}\right.$$

In Formula (48), $f^{non}\left(X\left(t\right),t\right)$ is the nonlinear function of state X(t), and

$$f^{non}\left(X\left(t\right),t\right)=(I-C_{n}^{n^{\prime }})\omega_{in}^{n}\left(t\right)+C_{b}^{n}\delta \omega_{in}^{b}\left(t\right)-C_{b}^{n}\delta \omega_{ib}^{b}\left(t\right),$$

$B^{non}$ is the input control matrix, $\Xi^{non}=\left[\begin{array}{ccc} 0_{3\times 3} & -C_{b}^{n^{\prime }} & 0_{3\times 3} \end{array}\right]$. ${\hat{X}}^{lin}\left(t\right)$ is the optimal estimation of the state of the linear part, it is also the control input of the nonlinear filter. $G^{non}\left(t\right)$ is the process noise control matrix of the nonlinear system, $G^{non}\left(t\right)=\left[-C_{b}^{n^{\prime }}\right]$. $w^{non}\left(t\right)$ is the noise vector, $w^{non}\left(t\right)∼N\left(0,Q{\left(t\right)}^{non}\right)$. $H^{non}\left(t+1\right)$ is the measurement matrix of the nonlinear system, $H^{non}\left(t\right)=\left[\begin{array}{cc} I_{3\times 3} & 0_{3\times 6} \end{array}\right]$. $v^{non}\left(t+1\right)$ is the measurement noise, $v^{non}\left(t+1\right)∼N\left(0,R^{non}\left(t+1\right)\right)$.

**Figure 2:**
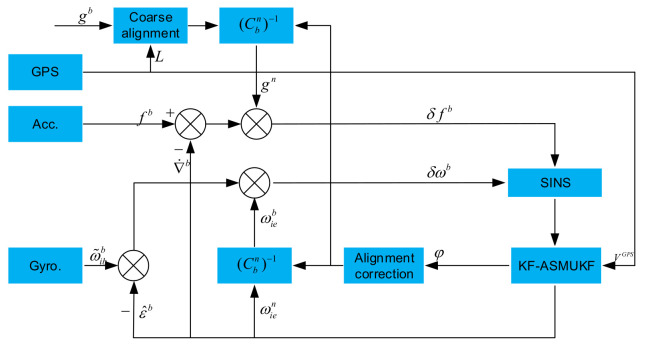
Flow chart of hybrid KF-ASMUKF filter alignment algorithm.

Based on the above analysis and derivation, the flow chart of the hybrid KF-ASMUKF alignment algorithm can be obtained, as shown in Figure 2:

Step 1: initialize the system, and apply the ASMUKF algorithm to the nonlinear partial filtering model at time t+1, as shown in Formula (48).

Step 2: through calculation, the best estimation of east-, north-, and up-misalignment angles can be obtained at time *t*.

Step 3: select the ASMUKF algorithm to perform filter estimation, ASMUKF one-step prediction and update refer to Formulas (31)–(42).

Step 4: obtain all state variable estimates according to filtering $\hat{x}\left(t\right)={\left[\begin{array}{cc} {\hat{x}}^{lin}\left(t\right) & {\hat{x}}^{non}\left(t\right) \end{array}\right]}^{T}$ and the value observed $Z^{lin}\left(t+1\right)$ at time t+1, then KF is applied to the linear partial filtering model as Formula (47), the estimation ${\hat{x}}^{\mathrm{li}n}\left(t+1\right)$ of the optimal state variable of the linear part at time it can be obtained. Finally, return to Step 1 to start the next cycle of filtering.

## 5. Experimental Results and Analysis

### 5.1. Simulation Test

**Figure 3:**
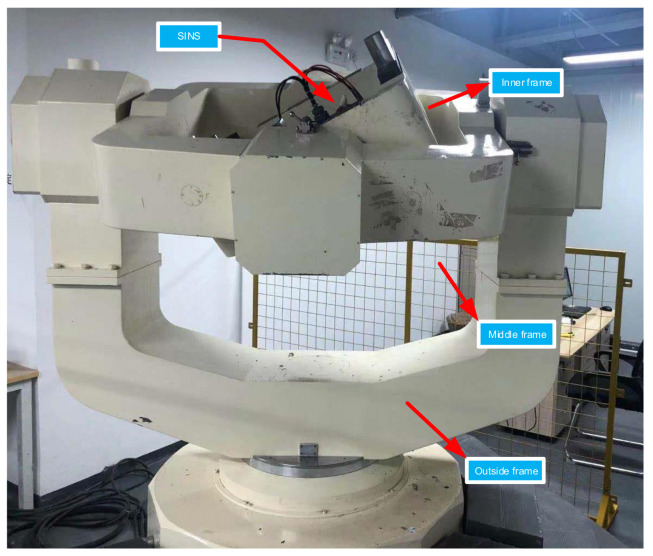
Three-axis, high-precision turntable.

To verify the effectiveness of the algorithm in this paper. We set up a swing experiment platform based on a fiber optic gyro strapdown inertial navigation system on the three-axis turntable in the laboratory to simulate the marine environment of the ship. The test equipment is shown in Figure 3.

The fiber optic strapdown inertial navigation system consists of three fiber optic gyroscopes with an accuracy of 0.01°/h and three flexible accelerometers with an offset of $5\times 10^{-4}$ g. The sampling frequency is 100 Hz. The three-axis turntable simulates the motion of the ship. The three-axis is set to sinusoidal signal. The local latitude and longitude is 24.6188° and the longitude is 118.0399°. the motion model for assessing the simulated ship’s pitch angle θ, roll angle γ and azimuth angle ψ:

(49) $$\begin{array}{c} \theta =\theta_{m}\sin\left(\pi t/5\right)\\ \gamma =\gamma_{m}\sin\left(\pi t/6\right)\\ \psi =\psi_{m}\sin\left(\pi t/8\right) \end{array}$$

In Formula (49), $\theta_{m}\;=\;4^{\circ }$, the swing period is 4 s, $\gamma_{m}\;=\;6^{\circ }$, the swing period is 6 s and $\psi_{m}\;=\;20^{\circ }$, the swing period is 10 s.

**Figure 4:**
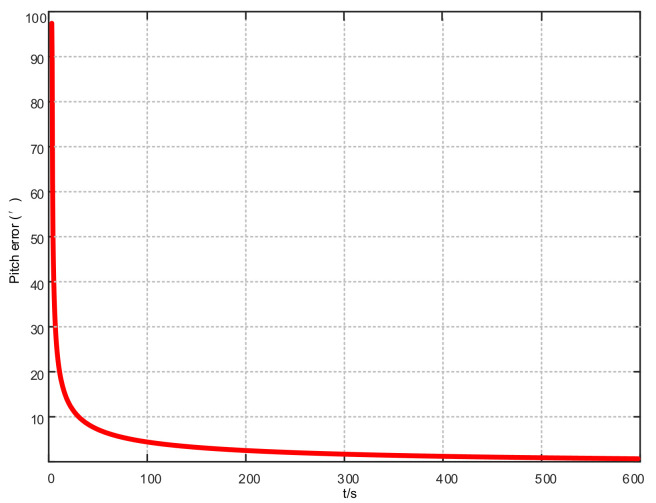
Pitch error results based on KF-ASMUKF hybrid filtering algorithm.

**Figure 5:**
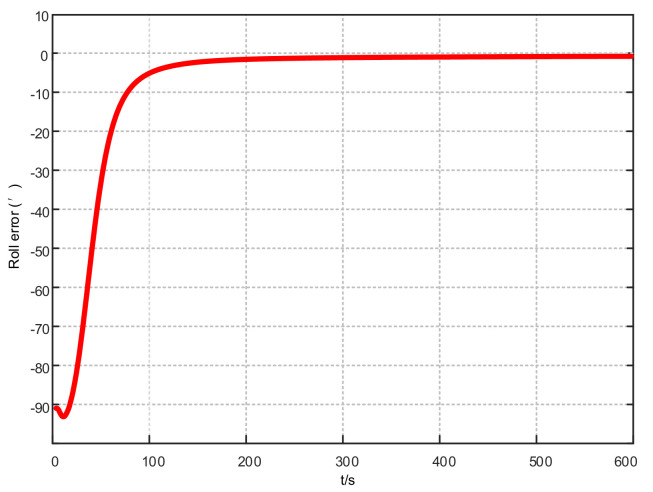
Roll error results based on KF-ASMUKF hybrid filtering algorithm.

**Figure 6:**
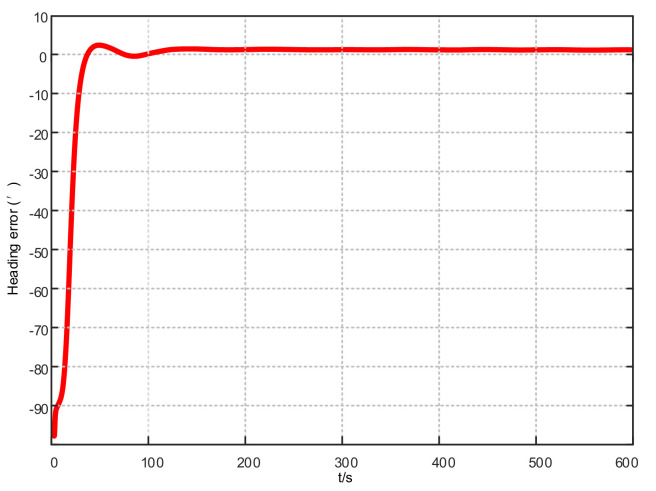
Heading error results based on KF-ASMUKF hybrid filtering algorithm.

Assuming that the system has completed coarse alignment, the hybrid filtering algorithm proposed in this article is used for fine alignment. The alignment results are shown in Figure 4, Figure 5 and Figure 6:

From Figure 4, Figure 5 and Figure 6, in the fine alignment stage of the KF-ASMUKF based hybrid filter algorithm, the pitch angle, roll angle, and heading angle converge faster, and the error is also small. The three attitude angle errors are about the first. After three minutes, it began to converge to a more accurate range.

**Figure 7:**
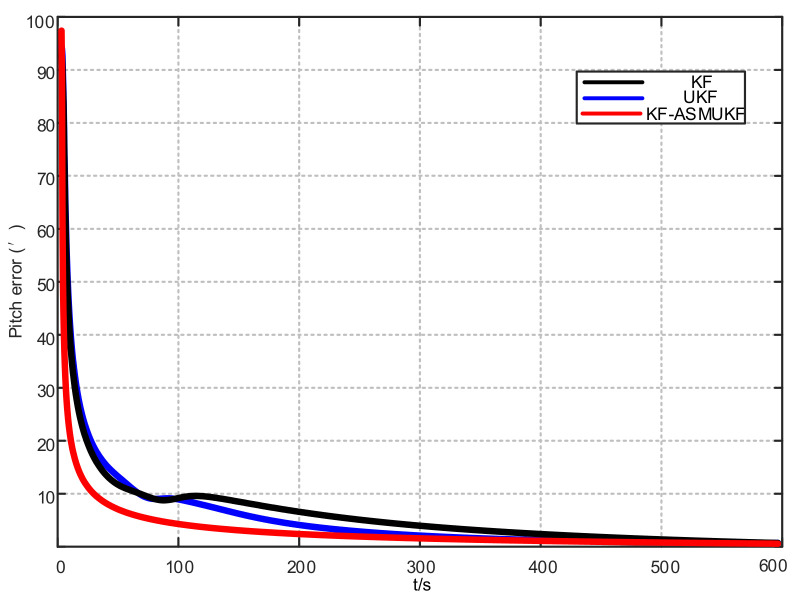
Pitch error results of different filtering algorithms.

**Figure 8:**
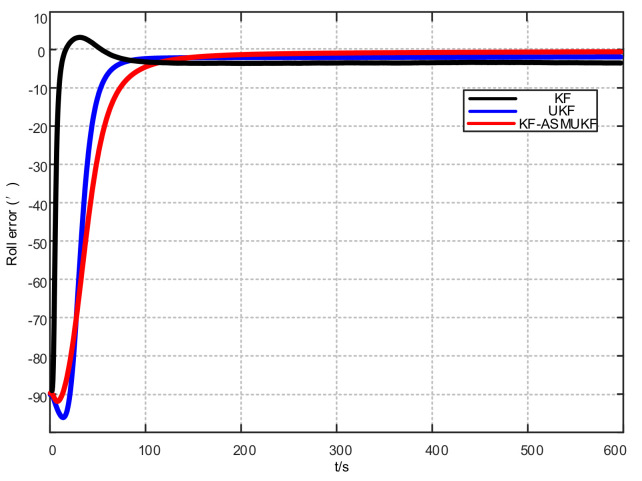
Roll error results of different filtering algorithms.

**Figure 9:**
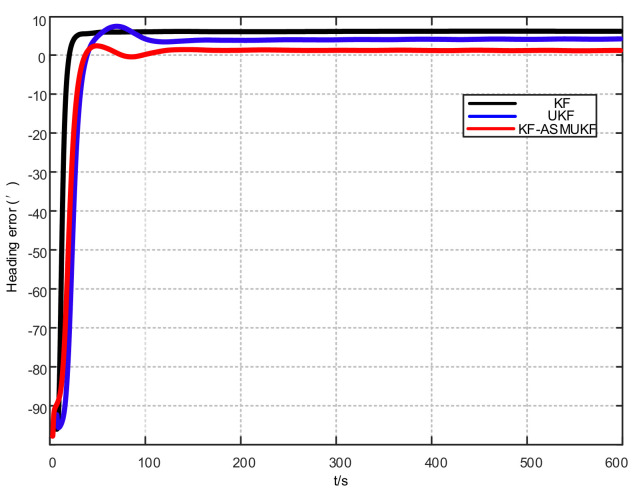
Heading error results of different filtering algorithms.

To further verify the correctness of the proposed hybrid filtering algorithm, four ship simulation mooring alignment tests were carried out in this paper. During the simulation test, the coarse alignment was 5 min. The fine alignment was 10 min, and the integrated navigation system was switched to integrated navigation after the alignment was completed. To facilitate the comparison of different filtering algorithms, the data after the fine alignment are intercepted respectively using KF, UKF, and KF-ASMUKF, then they perform the initial alignment in the mooring state. The alignment results are shown in Figure 7, Figure 8 and Figure 9 and Table 2:

According to Figure 7, Figure 8 and Figure 9 and Table 2, KF-ASMUKF can be used for SINS fine alignment. The attitude misalignment angle can quickly converge in a short time. The average of pitch error is less than 1.5${}^{\prime }$, the average of roll misalignment error is less than 2.5${}^{\prime }$, and the average heading error is less than 5${}^{\prime }$. The convergence process of the attitude misalignment angle estimation is stable and they have good stability. In addition, in this simulation environment, the estimated errors and standard deviations of the three attitude misalignment angles based on the KF-ASMUKF hybrid filter algorithm have certain advantages over KF and UKF, which verifies the effectiveness of the algorithm proposed in this paper.

### 5.2. Mooring Alignment Test on the Sea

**Figure 10:**
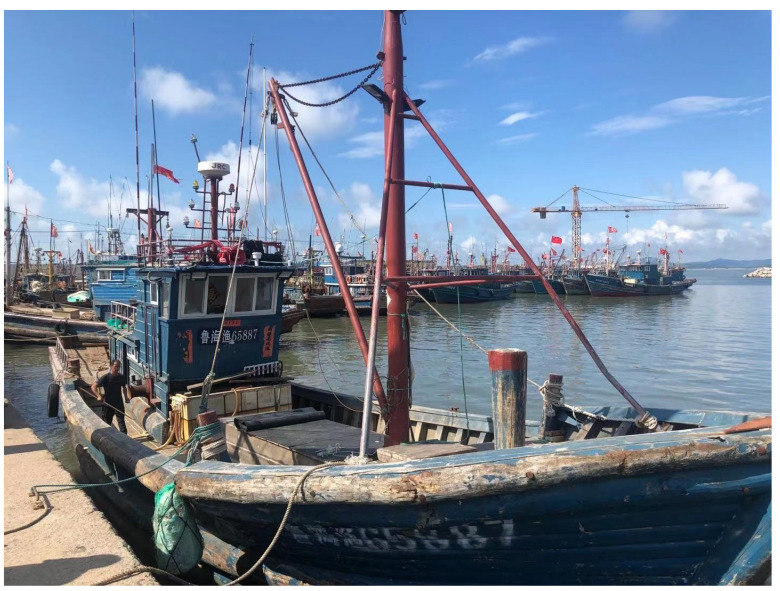
Ship of mooring alignment test.

**Figure 11:**
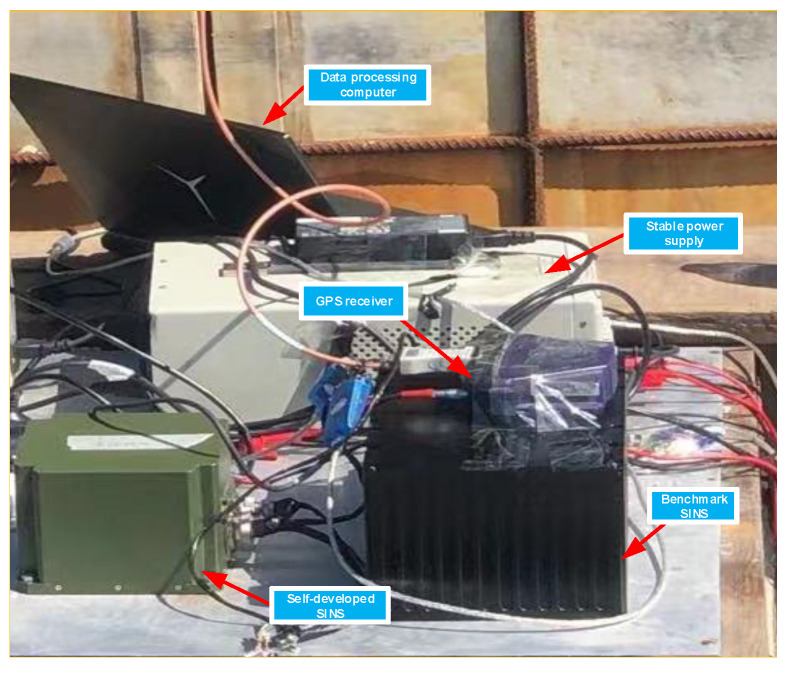
Related equipment required for test.

To further verify the effectiveness of the algorithm proposed in this paper, the ship’s alignment test under mooring conditions was carried out in Yantai, China. The ship used in this test is shown in Figure 10:

The test ship is equipped with a high-precision laser SINS integrated navigation system, and a self-made fiber optic SINS integrated navigation system, as shown in Figure 11:

On the test ship, the self-made fiber optic SINS-GPS integrated navigation system and the high-precision integrated navigation system are fixed using aluminum alloy plates. The self-made SINS can achieve a fast and precise alignment method, the high-precision integrated navigation system is used as the attitude reference datum. GPS is used to output speed and position information, and a stable power supply is used to power the inertial navigation system. Before the on-site power-on test, each group of SINS integrated navigation systems is accurately calibrated.

**Figure 12:**
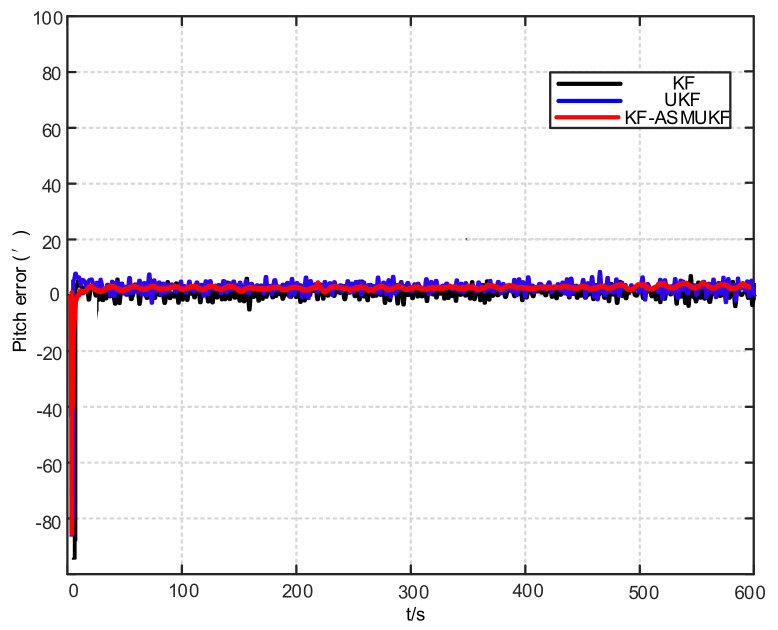
Pitch error results of different filtering algorithms.

**Figure 13:**
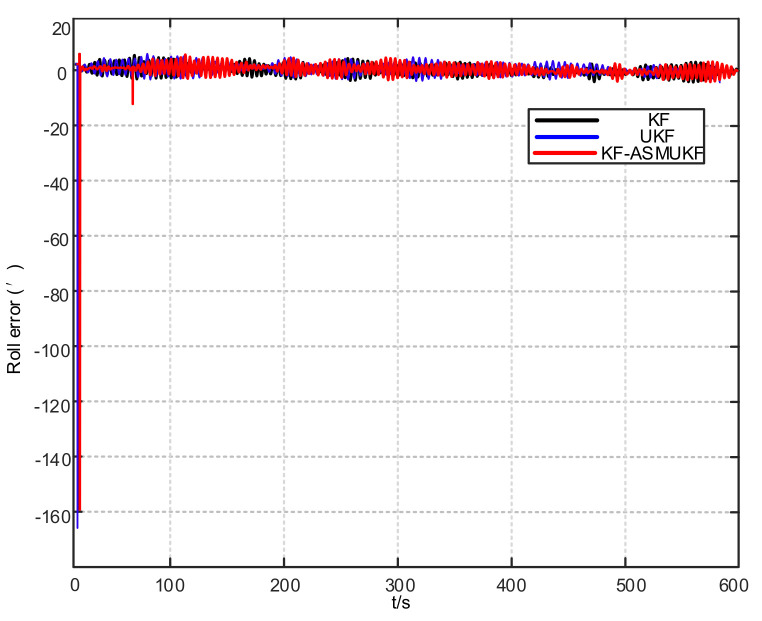
Roll error results of different filtering algorithms.

**Figure 14:**
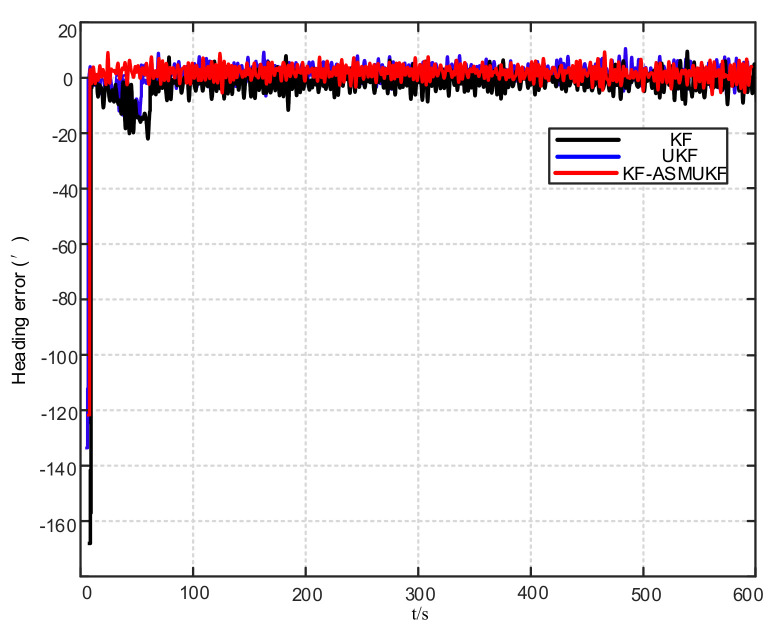
Heading error results of different filtering algorithms.

In this paper, four ship mooring alignment tests were carried out. During the ship test, the coarse alignment was 5 min and the fine alignment was 10 min. After the alignment was completed, it was converted to integrated navigation. To facilitate the comparison of different filtering algorithms, the data at the moment after the fine alignment are intercepted. The KF, UKF, and KF-ASMUKF based filtering algorithms are used to perform the initial alignment of the ship in the mooring state. The estimated attitude error results of different filtering algorithms are shown in Table 3 and Figure 12, Figure 13 and Figure 14:

According to Table 3 and Figure 12, Figure 13 and Figure 14, the proposed hybrid filtering algorithm based on KF-ASMUKF performs the initial alignment of ship’s SINS under mooring conditions. This method can make the three attitude misalignment angles fast in a short time. Convergence and the error are smaller than the KF algorithm and the UKF algorithm. The average of pitch error is 3.053${}^{\prime }$, the average of roll error is 3.332${}^{\prime }$, the average of heading error is 8.868${}^{\prime }$, which meets the alignment accuracy requirements of the ship’s SINS in the medium-precision mooring conditions. The estimation convergence process of the three attitude misalignment angles is stable, and they also have good stability. In addition, the estimated errors and standard deviations of the three attitude misalignment angles obtained based on the KF-ASMUKF hybrid filter algorithm have certain advantages over KF and UKF, which further verifies the effectiveness of the proposed algorithm.

## 6. Conclusions

Aiming at the problem of ship alignment under large azimuth misalignment under mooring conditions, a KF-ASMUKF hybrid filtering method is proposed. This paper deduced the ASMUKF algorithm, constructed its filter model, then combined it with the KF filter algorithm to form a KF-ASMUKF hybrid filter model through which the linear and nonlinear parts of the ship’s SINS alignment process were processed. This method reduces the computational complexity of the algorithm, improves the real-time performance of the filtering algorithm, and accelerates the convergence speed of mooring alignment under large azimuth misalignment angles. A simulation test of mooring alignment was carried out on a three-axis turntable, and an on-site mooring alignment test was carried out in Yantai. The experimental results show that this method improves the ship SINS alignment accuracy and reduces the amount of calculation in the ship SINS alignment process. It meets the accuracy requirements of medium-precision marine SINS, and lays the foundation for the later ship alignment and integrated navigation.

In addition, some future work needs to be improved and expanded:

(1) At present, the algorithm is carried out under relatively good sea conditions. In the future, the algorithm will be verified in a harsh environment.

(2) The algorithm was only tested for alignment in a mooring environment, and the effectiveness of the alignment algorithm will be verified during travel in the future. 

## Figures and Tables

**Table 1:** Algorithm flow based on ASMUKF.

**Algorithm:** ASMUKF filtering algorithm
**Input:**$x_{0}$, *R*, *Q*
**Output:** $x_{t/t}$
(1) Initialization: ${\hat{x}}_{0}$, ${\hat{x}}_{0}=E\left[x_{0}\right]$, $P_{0}=E\left[\left(x_{0}-{\hat{x}}_{0}\right){\left(x_{0}-{\hat{x}}_{0}\right)}^{T}\right]$
(2) **For t∈[1,n]**
(3) Calculate Sigma points and the weights of the mean and variance: $l_{i}$, $W_{i}^{m}$, $W_{i}^{c}$
(4) Time update: $\zeta_{t,i}=f\left(l_{t-1,i}\right)$, ${\hat{x}}_{t/t-1}$, $P_{x,t/t-1}$
(5) Measurement update: ${\hat{y}}_{t}$, $P_{y,t}$, $P_{xy,t}$
(6) Filter update: $K_{t}$, ${\hat{x}}_{t/t}$, $P_{x,t/t}$
(7) Calculate filter update sampling correction coefficient: $\alpha_{t+1/t}$, $\alpha_{t+1/t}=d_{t/t}/d_{\max}$. Return to step (2)
(8) **Return**: $x_{t/t}\leftarrow {\hat{x}}_{t/t}$
(9) **End for**

**Table 2:** Attitude error results of mooring alignment under different filtering algorithms (simulated environment).

	Pitch Error (${}^{\prime }$)		Roll Error (${}^{\prime }$)		Heading Error (${}^{\prime }$)	
	Mean	Std	Mean	Std	Mean	Std
KF	2.594	0.106	−3.752	0.125	7.342	0.235
UKF	2.284	0.062	−3.423	0.064	6.158	0.073
KF-ASMUKF	1.065	0.043	−2.251	0.032	4.599	0.041

**Table 3:** Attitude error results of mooring alignment under different filtering algorithms (real environment).

	Pitch Error (${}^{\prime }$)		Roll Error (${}^{\prime }$)		Heading Error (${}^{\prime }$)	
	Mean	Std	Mean	Std	Mean	Std
KF	5.615	0.023	5.522	0.224	12.276	0.429
UKF	4.477	0.022	4.450	0.251	10.246	0.392
KF-ASMUKF	3.053	0.015	3.332	0.101	8.868	0.285
